# Derivation of Chondrogenically-Committed Cells from Human Embryonic Cells for Cartilage Tissue Regeneration

**DOI:** 10.1371/journal.pone.0002498

**Published:** 2008-06-25

**Authors:** Nathaniel S. Hwang, Shyni Varghese, Jennifer Elisseeff

**Affiliations:** Department of Biomedical Engineering, Johns Hopkins University, Baltimore, Maryland, United States of America; Massachusetts Institute of Technology, United States of America

## Abstract

**Background:**

Heterogeneous and uncontrolled differentiation of human embryonic stem cells (hESCs) in embryoid bodies (EBs) limits the potential use of hESCs for cell-based therapies. More efficient strategies are needed for the commitment and differentiation of hESCs to produce a homogeneous population of specific cell types for tissue regeneration applications.

**Methodology/Principal Findings:**

We report here that significant chondrocytic commitment of feeder-free cultured human embryonic stem cells (FF-hESCs), as determined by gene expression and immunostaining analysis, was induced by co-culture with primary chondrocytes. Furthermore, a dynamic expression profile of chondrocyte-specific genes was observed during monolayer expansion of the chondrogenically-committed cells. Chondrogenically-committed cells synergistically responded to transforming growth factor-β1 (TGF-β1) and β1-integrin activating antibody by increasing tissue mass in pellet culture. In addition, when encapsulated in hydrogels, these cells formed cartilage tissue both *in vitro* and *in vivo*. In contrast, the absence of chondrocyte co-culture did not result in an expandable cell population from FF-hESCs.

**Conclusions/Significance:**

The direct chondrocytic commitment of FF-hESCs can be induced by morphogenetic factors from chondrocytes without EB formation and homogenous cartilage tissue can be formed *in vitro* and *in vivo*.

## Introduction

Articular cartilage is an avascular tissue that provides structural support and weight bearing function in diarthrodial joints. Functional loss of articular cartilage due to trauma or chronic disease may lead to debilitating conditions and osteoarthritis [1]. Tissue engineering is a multidisciplinary field of research that aims to regenerate damaged tissues and restore organ function through use of biomaterials and/or cells. Current therapies for cartilage repair are generally surgical including stimulation of bone marrow or autologous chondrocyte transplantation (ACT) with or without additional biomaterials [2], [3]. However, these strategies for repair often result in inferior cartilage tissue production, due in part to the limited proliferation and differentiation capability of the repair cells [4]. Therefore, there is a significant need to develop alternative cell sources that are easily cultured and are capable of yielding large cell numbers while retaining the capacity to differentiate and form cartilage tissue.

Bone marrow-derived mesenchymal stem cells (MSCs) have been investigated as candidate cells for musculoskeletal tissue regeneration applications including cartilage and bone [5]–[9]. MSCs have reached clinical testing, with potential repair mechanisms identified as direct tissue formation and/or secretion of trophic factors [10]. Even though MSCs are capable of multi-lineage differentiation, they can proliferate only to a limited degree and recent studies have suggested that cartilage engineered from MSCs may not be robust and stable in prolonged *in vivo* transplantation [11]–[14]. Human embryonic stem cells (hESCs), on the other hand, can proliferate significantly while still retaining the ability to differentiate into all three germ lineages [15]. Upon withdrawal of self-renewal factors, hESCs spontaneously differentiate towards numerous lineages [16]. A heterogeneous population of differentiated cells from hESCs may lead to inferior tissue function and organization of engineered tissues [16], [17]. Therefore, the potential use of hESCs for tissue engineering applications relies on the development of strategies to control and efficiently produce a homogenous cell population [18].

Chondrogenic differentiation of ESCs has previously been achieved by supplementation of growth factors such as bone morphogenetic protein-2, -4 or transforming growth factor-β1 [19]–[21]. We recently demonstrated that chondrocyte-secreted morphogenetic factors can promote the differentiation of mesenchymal cells and provide survival signals, resulting in enhanced expression of chondrocytic genes and ultimately cartilaginous nodule formation [22]. In the present study, we investigated hESC differentiation into a chondrocytic phenotype, without the formation of EBs, by co-culture with chondrocytes in the Transwell culture system. Our results indicate that Transwell co-cultured FF-hESCs expressed cartilage-specific Type II collagen and retained a chondrocyte phenotype during monolayer expansion. Moreover, when the chondrogenically-committed cells were encapsulated in poly(ethylene-glycol)-based hydrogels, they formed homogenous cartilage-like tissue *in vitro* and *in vivo,* without evidence of teratoma formation.

## Materials and Methods

### Feeder free culture of human embryonic stem cells

The BG02 hES cell line was obtained from Bresagen (Athens, GA) and cultured according to manufacturer's instructions. For feeder-free culture, hES cell cultures were dissociated into small clumps by incubating at 37°C for 30 minutes with 1 mg/ml collagenase IV (GIBCO, Gaithersburg, MD) and subsequently plated onto laminin-coated tissue culture plates and maintained with mouse embryonic fibroblast (MEF)-conditioned medium, as previously described [23], [24].

### Chondrocyte isolation and co-culture

For chondrocyte isolation, full thickness bovine cartilage (Research 87, Massachusetts) was harvested and cartilage pieces were digested in Dulbeco's Modified Eagle's Medium (DMEM, GIBCO, Grand Island, NY, U.S.A.) containing 0.2% collagenase (Worthington Biochemical Corporation, Lakewood, NJ, U.S.A.) and 5% fetal bovine serum (Hyclone) for 16 hours at 37°C, as previously described [25]. The cell suspension was then filtered through a 70 µm nylon filter (Cell Strainer; Falcon, Franklin Lakes, NJ, U.S.A.) and washed three times with Phosphate Buffered Saline (PBS) containing 100 U/ml penicillin and 100 µg/ml streptomycin. Isolated chondrocytes were seeded onto Transwell™ inserts (6 well plates) with a porous membrane (0.4 µm pore size) and lowered into the FF-hESCs wells for co-culture. Prior to co-culture, FF-hESCs were maintained in MEF-conditioned medium as colonies. Cells were co-cultured for 21 days in DMEM supplemented with 10% FBS, and 5000 U/mL penicillin and 5000 U/mL streptomycin, and 1 mM L-glutamine. Control FF-hESCs were maintained with DMEM supplemented with 10% FBS, and 5000 U/mL penicillin and 5000 U/mL streptomycin, and 1 mM L-glutamine. Medium was aspirated and exchanged twice a week. For co-culture experiments, chondrocytes were replenished at day 7 and day 14 with freshly isolated cells. After 3 weeks of co-culture, Transwell inserts with chondrocytes were removed. Chondrocyte-stimulated FF-hESCs were then trypsinized (0.25% trypsin/EDTA) and sequentially expanded at a seeding density of 2×10^4^ per cm^2^ in DMEM supplemented with 10% FBS, and 5000 U/mL penicillin and 5000 U/mL streptomycin, and 1 mM L-glutamine.

### Photoencapsulation

Chondrogenically-committed cells from hESCs (P8) were encapsulated into poly(ethylene glycol)-diacrylate (PEGDA) or tyrosine-arginine-glycine-aspartate-serine (YRGDS)-modified PEGDA hydrogels (PEG-RGD), as previously described [26]. Briefly, the PEGDA hydrogel solution was prepared by mixing 10% (w/v) PEGDA (SunBio Inc., Korea) in sterile phosphate-buffered saline (PBS) with penicillin (100 U/ml) and streptomycin (100 mg/ml, GIBCO). For PEG-RGD hydrogels, YRGDS was reacted with acryloyl-PEG-*N*-hydroxysuccinimide (acryloyl-PEG-NHS, 3400 MW; Nektar) in 50 mM TRIS buffer (pH 8.2) for 2 hours at room temperature. The product was lyophilized and the final concentration of the polymer solution (10% w/v) containing 2.5 mM RGD was prepared by mixing PEGDA and YRGDS-PEG-Acrylate in sterile PBS. Cells were gently mixed with the polymer solution at a concentration of 2×10^7^ cells/ml and photopolymerized with photoinitiator (Irgacure 2959, 0.05% w/v) using 365-nm light (4.5 mW/cm^2^) for 5 minutes. The constructs (total volume of 100 µl) were then cultured at 37°C with 5% CO_2_ in 2.5 ml chondrogenic differentiating medium for 3 weeks.

### Pellet culture

For pellet cultures, 2.5×10^5^ cells (P7) were collected in 1 ml Eppendorf tubes and centrifuged at ×150*g* for 5 minutes. Serum-free chondrogenic medium was prepared with DMEM (GIBCO) containing 2 mM L-glutamine, 100 nM dexamethasone, 50 µg/ml ascorbic acid phosphate (Wako, Neuss, Germany), 1 mM sodium pyruvate, 40 µg/ml proline, 1% ITS+ (Collaborative Biomedical Products, Bedford, MA) in the presence or absence of 10 ng/ml transforming growth factor (TGF)-β1. The effects of integrin activation were determined by incubation of pellets (n = 6) with blocking antibodies anti-β1 (Chemicon, Temecula, CA) or activator anti-β1 antibody (Chemicon).

### Cell proliferation

Growth kinetics of cells were monitored by plating at densities ranging 2500 cells/cm^2^ to 2×10^4^ cells/cm^2^ in 6-well tissue culture plates. Cells were serially passaged using trypsin/EDTA (Invitrogen) and counted using a Z2 Coulter Particle Counter.

### Reverse transcriptase-PCR and real-time PCR

Total RNA was extracted with Trizol. Two micrograms of total RNA per 20 µl of reaction volume were reverse-transcribed into cDNA using SuperScript First-Strand Synthesis System (Invitrogen). Real Time-PCR reactions were performed using the SYBR Green PCR Mastermix and the ABI Prism 7700 Sequence Detection System (Perkin Elmer/Applied Biosystems, Rotkreuz, Switzerland). cDNA samples (2 µl for total volume of 25 µl per reaction) were analyzed for gene of interest and for the reference gene β-actin. The level of expression of each target gene was then calculated as –2^ΔΔCt^ as previously described [27]. Each sample was repeated at least three times for the gene of interest. The PCR primers are listed in Table 1.

**Table 1:** Primer Sequences

Gene	Primer Sequences (5′-3′)	Predicted size (bp)
AGN	F-tgaggagggctggaacaagtacc	350
	R-ggaggtggtaattgcagggaaca	
Col I	F-tgacgagaccaagaactg	600
	R-ccatccaaaccactgaaacc	
Col II	F-tttcccaggtcaagatggtc	377
	R-cttcagcacctgtctcacca	
Nestin	F-gccctgaccactccagttta	200
	R-ggagtcctggatttccttcc	
flk-1	F-ggtattggcagttggaggaa	199
	R-acatttgccgcttggataac	
myf5	F-tcacctcctcagagcaacct	193
	R-tgaagccttcttcgtcctgt	
ALP	F-acgtggctaagaatgtcatc	476
	R-acatttgccgcttggataac	
Col X	F-ccctttttgctgctagtatcc	468
	R-ctgttgtccaggttttcctggcac	
COMP	F-caggacgactttgatgcaga	314
	R-aagctggagctgtcctggta	
link protein	F-gctctgtgcaatatcccatc	232
	R-cccacttttgcaatgtgagc	
Sox-9	F-acgtcatctccaacatcgagacc	4454
	R-ctgtagtgtgggaggttgaaggg	
Cbfa1	F-ccacccggccgaactggtcc	258
	R-cctcgtccgctccggcccaca	
BMP4-RII	F-tagtcactgacaacaacggtgcagtc	816
	R-tatactgctccatatcgacctcggc	
TGF-B-RII	F-tagtcactgacaacaacggtgcagtc	539
	R-acagtgctcgctgaactccatgagc	
TERT	F-agctatgcccggacctccat	185
	R-gcctgcagcaggaggatctt	
β-actin	F-tggcaccacaccttctacaatgagc	396
	R-gcacagcttctccttaatgtcacgc	

### Subcutaneous transplantation into athymic nude mouse

Animals were anesthetized via intraperitoneal injection of 7.5 mg/kg ketamine, 0.24 mg/kg acepromazine and 1.5 mg/kg xylazine. Chondrogenically-committed cells (P8) encapsulated in PEGDA and PEG-RGD hydrogels (n = 6) were implanted subcutaneously into the dorsal region of 6 to 8-week-old athymic mice for 12 and 14 weeks. Harvested constructs were processed for biochemical analysis. In addition, constructs were fixed in 4% paraformaldehyde for 24 hours at 4°C and further processed for histology and immunohistochemistry.

### Biochemical assays

Biochemical assays were performed on hydrogel constructs harvested from animals after 12 and 24 weeks. Constructs (n = 6) were digested in papainase solution (construct/1 ml papainase solution; 125 µg/mL, Worthington Biomedical, Lakewood, NJ) for 16 hours at 60°C. The DNA content was determined by Hoescht 33258 dye assay, as previously described [7]. The GAG content was quantified using dimethylmethylene blue (DMMB) spectrophotometric assay at A_525_, as previously described [28]. Total collagen content was determined by measuring the hydroxyproline content of the constructs after acid hydrolysis and reaction with *p*-dimethylaminobenzaldehyde and chloramine-T, as previously described [29].

### Histology, immunostaining, and immunohistochemistry

For immunostaining, cells were fixed in 4% paraformaldehyde, rinsed with PBS and permeated with 0.1% Triton in PBS. Cells were blocked with 5% normal goat serum in PBS for 30 minutes, and incubated with rabbit polyclonal antibodies against Types I and II collagen (RDI, Flanders, NJ), Oct-4, SSEA-4, and Tra-180 (Chemicon) with 1∶100 dilutions. Cells were incubated with either FITC- or Texas Red- conjugated goat anti-rabbit secondary antibody (all 1∶100 dilutions) for 1 hour (Jackson ImmunoResearch laboratory, West Grove, PA). Nuclei were counterstained with DAPI (Chemicon) for 10 minutes. Images were collected with a Zeiss LSM Metal Confocal microscope. For histological analysis, constructs were fixed in 4% paraformaldehyde, dehydrated in serial ethanol dilutions, and paraffin embedded. Constructs were then cut into 5 µm sections and stained with Safranin-O/fast green or hematoxylin and eosin. Immunohistochemistry for Types I, II, and X collagen were performed as previously described [19].

### Statistical analysis

Data are expressed as mean±standard deviation (SD). Statistical significance was determined by analysis of variance (ANOVA single factor) with *P<0.05 or **P<0.01.

## Results

### Feeder free culture of human embryonic stem cells

**Figure 1:**
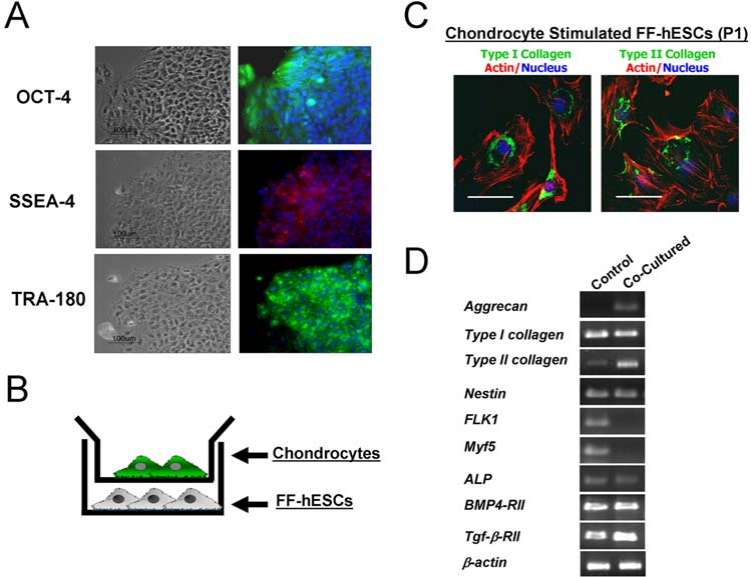
Derivation of chondrogenically-committed cells from FF-hESCs. (A) FF-BG02 hESCs maintained with MEF-conditioned medium supplemented with 4 ng/ml bFGF (bar = 100 µm) expressed Oct-4, SSEA-4, and Tra-180. (B) FF-hESCs were then co-cultured with chondrocytes in Transwell tissue culture for 3 weeks. (C) Upon stimulation, fibroblastic cells appeared (as indicated by actin phalloidin staining, red) and stained positive for both Type I collagen and Type II collagen (bar = 20 µm). (D) RT-PCR confirmed that chondrocyte co-culture resulted in upregulation of cartilage-specific markers including aggrecan, Type II collagen, and TGF-β-RII gene expressions and decreased expression of FLK1 and Myf5.

The BG02 human embryonic stem cell (hESC) line was maintained feeder free by culturing on laminin-coated culture dishes with mouse embryonic fibroblast (MEF)-conditioned hESC medium, supplemented with 4 ng/ml bFGF [23]. Feeder free-conditioned hESCs (FF-hESCs) proliferated in colonies, and were capable of forming embryoid bodies (EBs) (data not shown). FF-hESCs retained the expression of the pluripotent marker Oct-4, a POU domain transcription factor exclusively expressed in undifferentiated hESCs (Figure 1A). In addition, SSEA-4 and TRA-180 were also detected via immunostaining (Figure 1A).

### Generation of chondrogenically-committed cells

Recent studies suggest that morphogenetic factors from chondrocytes can induce chondrogenic differentiation of mesenchymal progenitor cells and hESCs [22], [30]. To investigate whether chondrocyte-secreted morphogenetic factors can stimulate the chondrogenic commitment of hESCs without EB formation, FF-hESCs were co-cultured with primary chondrocytes for 3 weeks using a Transwell tissue culture system (Figure 1B). After 3 weeks of co-culture, FF-hESCs were further expanded in monolayer culture. Upon initial replating, the adherent cell population displayed fibroblastic morphology. Immunostaining indicated that the chondrocyte-stimulated cells expressed both Type I collagen and Type II collagen (Figure 1C). RT-PCR analysis indicated that chondrocyte co-cultured FF-hESCs upregulated Type II collagen and aggrecan gene expression while inhibiting the expression of hematopoietic and myogenic genes such as FLK1 and Myf5. In addition, chondrocyte co-cultured FF-hESCs upregulated the expression of TGF-β1-RII with no significant upregulation of the osteogenic markers ALP and BMP4-RII genes, suggesting that co-culture resulted in chondrocytic differentiation of FF-hESCs (Figure 1D). In contrast, control FF-hESCs that were maintained in DMEM (10% FBS) for 3 weeks without the chondrocyte co-culture expressed multiple lineage markers, indicating heterogeneous differentiation of the hESCs.

### Characterization of chondrogenic-committed cells from hESCs

**Figure 2:**
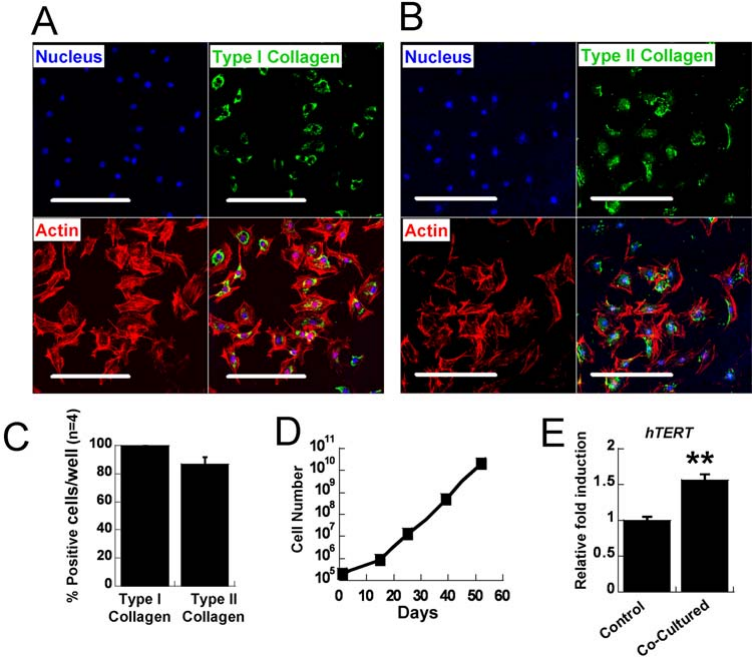
Characterization of chondrogenically-committed cells. At P1 most of the cells stained positive for both Types I and II collagen. (A) Immunostaining of Type I collagen and (B) Type II collagen at P1. (C) Quantification of cells positive for Types I and II collagen at P1 (n = 4 wells). (D) Proliferation rate of the chondrogenic-committed cells was measured by counting the cells during expansion. (E) At P1, chondrogenic-committed cells showed enhanced expression of telomerase gene compared to that of hESCs that were not cocultured (at P1). (** P<0.01, bar = 100 µm)

**Figure 3:**
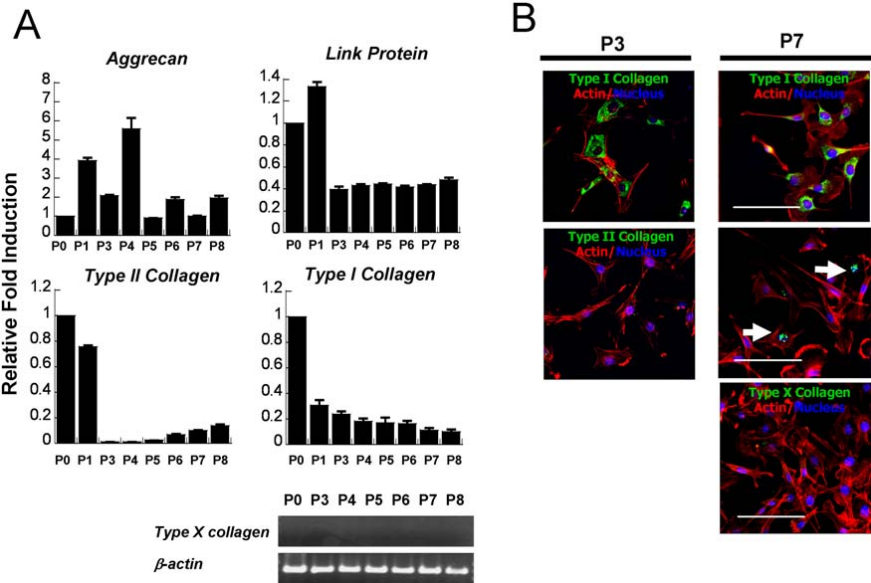
Expansion of chondrogenic-committed cells *in vitro*. (A) Dynamic expression profile of chondrocyte-specific genes observed during the expansion. Note that coculture-stimulated Type II collagen gene expression present at P0 and subsequently lost at P3 and P4. However, these cells slowly regained the expression of Type II collagen upon expansion (P5-8). (B) Immunostaining for Types I and II collagens confirmed real-time PCR. Note Type X collagen was not detected at any time. (bar = 20 µm)

We further characterized the chondrogenic-committed cells from FF-hESCs in monolayer. Upon initial plating of the chondrogenically-committed cells, 100% of the cells stained positive for Type I collagen and approximately 85% of the cells were Type II collagen positive (Figure 2A, B, and C). Proliferation was assessed by initially plating the cells at a density of 2×10^4^ cells/cm^2^ and monitoring cell number at confluence. Chondrocyte co-culture resulted in an expandable cell population from FF-hESCs with increased telomerase activity compared to that of control FF-hESCs (Figure 2E). Chondrogenically-committed cells initially displayed a low proliferation rate, however proliferation increased significantly after subsequent passaging (Figure 2D). The gene expression profile of chondrogenically-committed cells showed a dynamic expression of chondrocyte-specific markers (Figure 3A). Upon co-culture, chondrogenically-committed cells expressed high levels of Type II collagen; however, these cells rapidly lost Type II collagen gene expression upon expansion. At P3, these cells produced no detectable expression of Type II collagen by RT-PCR (data not shown). However, the cells regained Type II collagen gene expression, and reduced Type I collagen gene expression, upon further expansion (Figure 3A). Aggrecan and link protein were highly expressed at early passages and retained their expression throughout culture. Type X collagen, a hypertrophic chondrocyte marker, was not found in any of the cultures either by RT-PCR or immunostaining. Immunostaining for Types I and II collagen confirmed the real time-PCR results (Figure 3B). At P3, Type II collagen was not detected, however Type II collagen positive cells were found at P7 (Figure 3B).

### Effects of TGF-β1 and β1-integrin on cartilaginous tissue development

**Figure 4:**
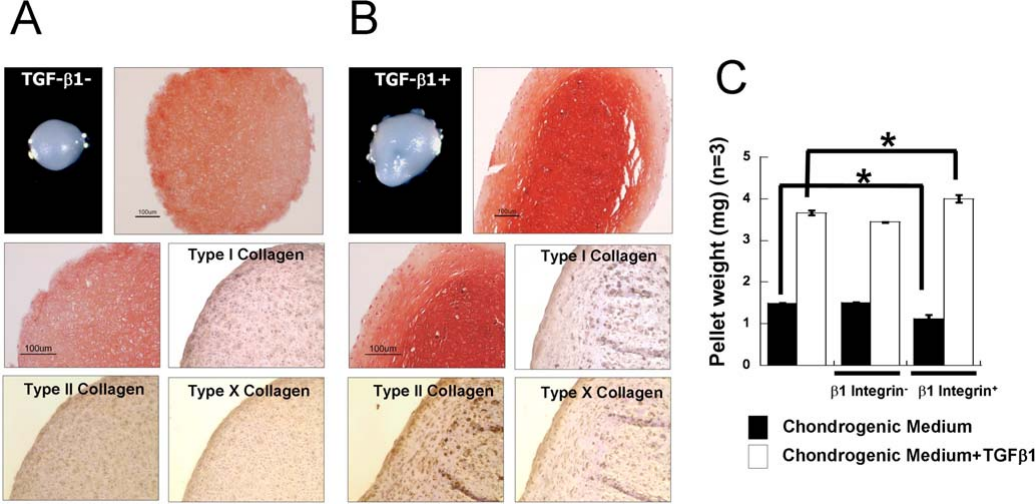
TGF-β1 and β1-integrin activation synergistically enhanced pellet weight. Chondrogenic-committed cells were pelleted and cultured in chondrogenic medium supplemented with or without TGF-β1 (10 ng/ml) for 3 weeks. (A) Safranin-O staining and Type I, Type II, and Type X collagen immunohistochemistry of pellets cultured without TGF-β1. (B) Safranin-O staining and Type I, Type II, and Type X collagen immunohistochemistry of pellets cultured in the presence of TGF-β1. (C) Pellets were weighed after 3 weeks of culture, with or without β1-integrin activating antibody or blocking antibody (n = 5). (* P<0.05, bar = 100 µm)

The pellet culture system is a standard method to screen three dimensional cartilage forming capacity [21], [31]. To examine the cartilage tissue forming abilities of chondrogenic-committed cells, pellets were formed and cultured for 3 weeks in chondrogenic medium in the presence or absence TGF-β1 (10 ng/ml). Initial gross observation of the pellets after 3 weeks indicated that TGF-β1 stimulated matrix production and cartilage-like tissue formation (Figure 4A). Supplementation with TGF-β1 in the medium resulted in a 245% increase in pellet weight (Figure 4C). However, chondrogenic-committed cells in pellets, regardless of TGF-β1 supplementation, displayed a spherical morphology typical of chondrocytes and were surrounded by an abundant extracellular matrix with glycosaminoglycans and Type II collagen, as detected by Safranin-O staining and immunohistochemistry for Type II collagen, respectively (Figure 4A, B). In contrast, Type I collagen and Type X collagen were detected with less intensity (Figure 4A, B).

We further investigated whether activation of β1-integrin plays a role in chondrogenic differentiation and cartilage tissue formation (Figure 4C). β1-integrin activating antibody or blocking antibodies were supplemented in the medium and pellets were cultured for 3 weeks. In the presence of TGF-β1, activation of β1-integrin resulted in increased pellet weight; while in the absence of TGF-β1, activation of β1-integrin resulted in slightly lower weight compared to control pellets. Inhibition of β1-integrin activity with blocking antibody in the presence of TGF-β1 also resulted in reduced pellet weight. In the absence of TGF-β1, blocking β1-integrin did not stimulate pellet weight. Activation of β1-integrin in the presence of TGF-β1 synergistically enhanced matrix production; however β1-integrin stimulation in the absence of TGF-β1 may be compromised.

### *In vitro* cartilage development in hydrogels

**Figure 5:**
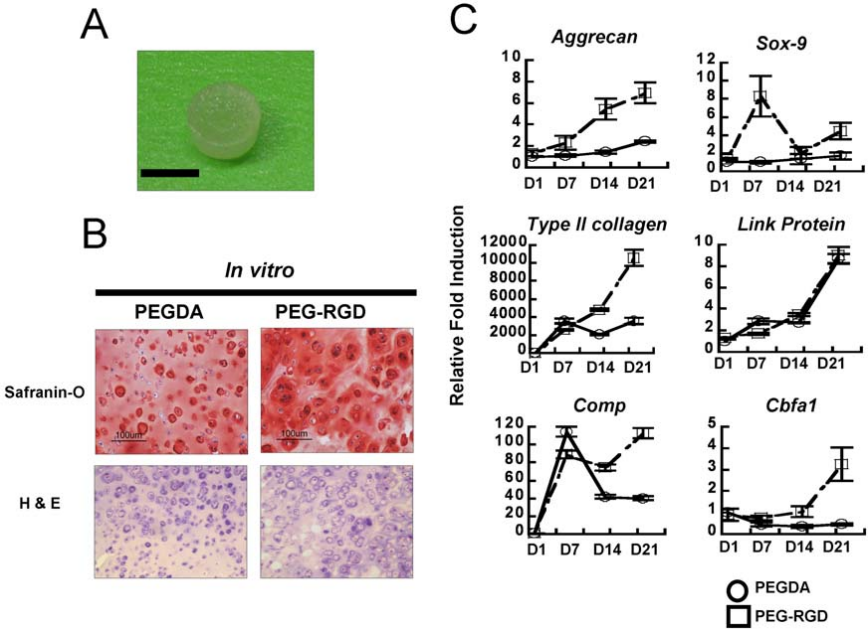
(A) Gross image of a poly(ethylene glycol)-diacrylate (PEGDA) hydrogel. (bar = 5 mm) (B) Safranin-O and H&E staining of chondrogenic-committed cells in PEGDA or PEG-RGD hydrogels after 3 weeks *in vitro* culture. (bar = 100 µm) (C) Real-time PCR analysis indicated distinct cellular response in RGD-modified hydrogels.

The pellet is a useful screening system to evaluate the chondrogenic potential of cells; however it does not yield the large tissue volumes that are needed in clinical settings of cartilage loss. Our laboratory and others have developed a PEG-based photopolymerizing hydrogel that can be implanted in clinically-relevant articular cartilage defects. The hydrogel system also supports simple polymer modification with integrin binding peptides such as tyrosine-arginine-glycine-aspartate-serine (YRGDS) [32]–[35]. These hydrogels are hydrophilic, biocompatible scaffolds that allow diffusion of nutrients and signaling molecules to support formation of large tissue volumes (Figure 5A). We therefore investigated the *in vitro* cartilaginous tissue forming abilities of chondrogenic-committed cells (P8) encapsulated in PEGDA or YRGDS-modified PEG-based hydrogels (PEG-RGD) hydrogels. Cell-laden hydrogels were maintained in chondrogenic medium containing TGF-β1 for 3 weeks. PEG-RGD hydrogels were utilized to provide adhesion sites for β1-integrins for stimulation similar to the pellet cultures. Histological analysis of both hydrogels indicated the formation of cartilage-like tissues as indicated by the presence of basophilic ECM components (Figure 5B). Cells in the PEG-RGD hydrogels produced greater Safranin-O staining, indicating more proteoglycan secretion. In addition, PEG-RGD hydrogels stimulated the expression of cartilage specific genes compared to the cells in the PEGDA hydrogels (Figure 5C). Throughout the *in vitro* culture, aggrecan, Type II collagen, Sox-9, and Cbfa1 genes were highly expressed in PEG-RGD hydrogels in contrast to cells in the PEGDA hydrogels. Gene expression of cells in the PEG-RGD hydrogels mimicked aspects of the early mesenchymal condensation process, and chondrogenically-committed cells with Sox-9 expression peaking at day 7, followed by upregulation of Type II collagen and Comp genes.

### Chondrogenic-committed cells from hESCs maintain chondrocyte phenotype in long-term *in vivo* transplantation

**Figure 6:**
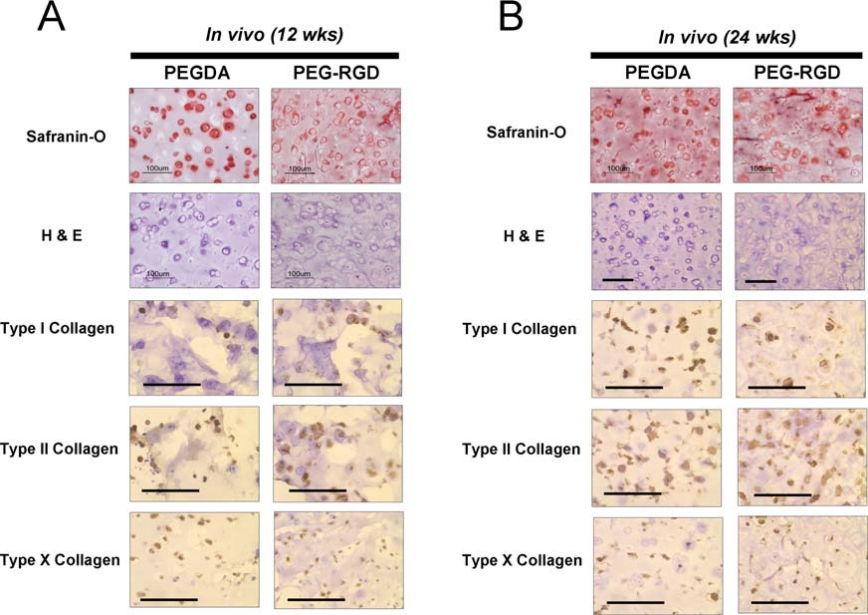
Histological analysis of *in vivo* engineered cartilage tissue. Chondrogenic-committed cells (P8) were encapsulated in PEGDA or PEG-RGD hydrogels and subcutaneously transplanted into athymic nude mice for (A) 12 weeks and (B) 24 weeks. Basophilic extracellular matrix deposition was observed and cells stained positive for Types I, II, and X collagen at both time points. (bar = 100 µm)

**Figure 7:**
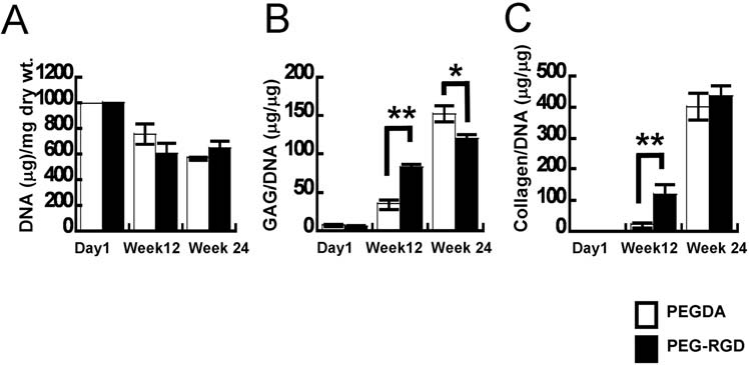
Biochemical analysis of *in vivo* engineered cartilage tissues. Quantification of DNA and extracellular matrix production was determined by biochemical assays. (A) DNA content was quantified with Hoechst 33258. (B) GAG content quantified by DMMB assay and normalized to the total DNA of the construct. (C) Collagen was quantified by measuring hydroxyproline content and normalized to the total DNA of the construct. (*P<0.05 or **P<0.01)

To validate the capacity of chondrogenically-committed cells to maintain chondrocyte phenotype *in vivo*, expanded cells (P8) were transplanted into athymic nude mice in PEGDA or PEG-RGD hydrogels, without exogenous growth factors. No evidence of teratoma formation was observed in any of the tissue constructs. Histological examination of the cells revealed cartilage tissue formation in both hydrogels at 12 weeks and 24 weeks (Figure 6A, B). Cells in both hydrogels were surrounded by Safranin-O-positive extracellular matrix. Immunohistochemistry at 12 and 24 weeks demonstrated an ECM in both hydrogels comprised primarily of Type II collagen, with Types I and Type X collagen also present (Figure 6A, B). Biochemical analyses indicated similar cellularity in both hydrogels and an extracellular matrix comprised of proteoglycans and collagens (Figure 7A, B, C). Greater amounts of GAG and collagen were observed in PEG-RGD hydrogels compared to PEGDA hydrogels at 12 weeks. However, after 24 weeks, more GAG accumulated in PEGDA hydrogels, with comparable amounts of collagen detected in both hydrogels.

## Discussion

The success of hESC-based cartilage tissue engineering depends in part on the development of efficient strategies to control cell differentiation along with tissue production and maintenance. Conventional methods to differentiate hESCs include EB formation. However, EBs generally contain a mixture of cells, differentiated into multiple lineages, highlighting the challenge of achieving a homogenous cell population [36]. To circumvent the production of a heterogenous EBs, we utilized FF-hESCs for direct differentiation into a chondrocytic lineage, devoid of EB formation. Chondrocyte-secreted morphogenetic factors were harnessed to create a more uniform microenvironment to stimulate chondrogenic commitment without the addition of exogenous growth factors. Chondrogenic-committed cells derived from FF-hESCs were identified by Type II collagen immunostaining and expression of chondrocyte-specific genes. Three weeks of chondrocyte co-culture resulted in a significant number of FF-hESC-derived cells expressing Type II collagen. In addition, RT-PCR analysis demonstrated that the chondrocyte co-culture resulted in the upregulation of chondrocyte specific markers in the FF-hESCs and decreased markers of other lineages. Critical for engineering tissues, the co-cultured cells maintained a chondrocyte phenotype *in vitro* and *in vivo*.

In this study, we utilized morphogenetic factors secreted from bovine articular chondrocytes to provide the commitment signals. The exact molecule(s) responsible for differentiation of the hESCs still remains a question. Our initial screen of growth factors indicated that differentiation was independent of the TGF-beta family-mediated signaling pathway (data not shown). Recently Kim et al., identified a novel molecule (Cytokine-like 1, Cytl1) secreted by chondrocytes which regulated multiple steps of mesenchymal condensation and chondrogenesis [37]. Cytl1 expression was strongly correlated with chondrocyte differentiation and was mainly expressed in terminally differentiated chondrocytes both *in vivo* and *in vitro* [37]. Exogenous Cytl1 and overexpression of Cytl1 demonstrated the capacity of this protein to induce chondrogenic differentiation of mesenchymal cells while preventing hypertrophy [37]. In addition, a study by D'Angelo et al., found that co-culture of articular chondrocytes with mesenchymal cells resulted in reduced hypertrophy [38]. Paracrine factors, secreted by chondrocytes, interacted with the stem cells, leading to direct expression of cartilage specific markers and prevention of further differentiation into heterotypic or hypertrophic tissues. Elucidation of the molecular signals stimulated by chondrocyte-secreted factors would lead to a better understanding of chondrogenic commitment of hESCs and likely improve hyaline cartilage tissue engineering efforts.

In the present study, cells were cocultured in DMEM containing 10% FBS. Cocultured FF-hESCs exhibited increased viability and eventually a significant proliferative potential. This observation is in accordance with previous reports where morphogenetic factors from chondrocytes enhanced the survival of mesenchymal cells and stimulated chondrogenic differentiation [22], [39]. However, culture in DMEM with 10% FBS medium in the absence of coculture resulted in heterogeneous differentiation of FF-hESCs after 3 weeks. In addition, FF-hESCs in DMEM with 10 % FBS resulted in greater cell death and cell senescence after trypsinization (0.25% Trypsin/EDTA).

To date, the chondrogenic differentiation of ESCs has required the addition of exogenous growth factors such as TGF-β1 or BMP-2 [19], [20], [40]. A recent study by Vats et al., suggested that the chondrogenic differentiation of hESCs can be achieved by co-culture with chondrocytes, without exogenous growth factors [30]. Similarly, we demonstrated that chondrocyte co-culture can result in chondrogenically-committed cells, with approximately 85% of cells staining positive for Type II collagen, without exogenous addition of growth factors. Chondrogenic-committed cells derived from FF-hESCs behaved similar to primary chondrocytes, exhibiting characteristics of dedifferentiation during early expansion [41]. We speculate that initial coculture resulted in coordinated signaling between cells, resulting in morphogenetic factor-mediated stimulation and production of Type II collagen, but rapid phenotype loss upon expansion in DMEM with 10% FBS medium. Expansion of the chondrogenic-committed cells in chondrocyte-conditioned medium delayed the dedifferentiation process (Figure S1). However, upon further expansion, the chondrogenic-committed cells regained their phenotype with Type II collagen expression. An elucidation of pathways regulating the phenotypic stability of chondrogenic-committed cells may provide strategies for efficiently generating more stable cells for cartilage repair using hESCs.

Designing biomaterials that stimulate ECM-cell interactions is an important component of utilizing stem cells for tissue engineering [42]. A number of studies have suggested that inducing ECM-cell interactions promotes cell differentiation and tissue formation [43], [44]. Specifically, recent evidence suggests β1-integrin is a key membrane receptor for relaying ECM-mediated signals via activating FAK and downstream signals, resulting in enhanced matrix accumulation and tissue formation [45]. Our study also implies that chondrogenic-committed cells responded to β1-integrin activation, but only in the presence of TGF-β1. Blocking β1-integrin activity resulted in reduced tissue mass. The *in vitro* study with RGD-modified hydrogels confirmed that the presence of ECM-cell interactions through integrin binding resulted in activation of cartilage specific markers. Histologically, cells in PEG-RGD hydrogels displayed stronger Safranin-O staining compared to the cells in PEGDA hydrogels.

Quality of tissue formed and long-term *in vivo* stability are important parameters to consider when contemplating clinical relevance and translation. Our *in vitro* study showed that chondrogenic-committed cells formed robust cartilage-like tissue in the pellet culture system as well as porous biomaterial scaffolds *in vitro* (Figure S2). In order to observe phenotypic stability of chondrogenic-committed cells *in vivo*, we utilized PEG-based hydrogels as a carrier. Long-term *in vivo* subcutaneous transplantation of cells in PEG-based hydrogels indicated that the chondrogenic-committed cells maintained phenotype and tissue quality. Cells encapsulated in PEG-RGD hydrogels responded by producing 240% more GAG and 595% more collagen at 12 weeks compared to that of cells encapsulated in PEGDA. However, after 24 weeks of transplantation, similar amounts of collagen and higher amounts of GAG accumulated in PEGDA hydrogels compared to PEG-RGD hydrogels. Hyaline cartilage is composed mainly of Type II collagen. However, chondrogenic-committed cells in both hydrogels displayed similar levels of Types I and II collagen, indicating some fibro-cartilaginous tissue formation in the subcutaneous space.

The overall goal of this study was to investigate the potential of differentiating hESCs into chondrogenically-committed cells without the EB formation. Specifically, we designed a system to differentiate hESCs without growth factors and produce cartilage-like tissue that macroscopically and phenotypically resembled native cartilage from FF-hESCs.

## Supporting Information

Figure S1 Chondrogenically-committed cells dedifferentiated upon initial expansion. At P3, chondrogenically-committed cells mainly expressed type I collagen (A) and type II collagen (B) was minimally detected when cultured with expansion medium (DMEM/FBS 10%). However, expansion of the cells in chondrocyte conditioned medium resulted in greater number of type II collagen positive cells (C, D). (E) Quantification of type II collagen positive cells that were expanded with either chondrocyte-conditioned medium (CM) or control expansion medium (C).
(2.30 MB TIF)

Figure S2 Safranin-O staining with eosin counter staining of in vitro cartilage tissue formation with chondrogenically-committed cells seeded on 3-dimensional porous scaffolds. Three dimensional porous scaffold composed of poly-(L-lactic acid) (PLLA) and poly-(lactic-glycolic acid) (PLGA) were fabricated by initially dissolving PLLA/PLGA 1:1 in chloroform to yield a solution of 5% (wt/vol) polymer. 0.25 ml of polymer solution was loaded into molds packed with 0.4 g of sodium chloride particles. The solvent was allowed to evaporate overnight and the sponges were subsequently immersed for 12 h in distilled water (changed every 2 hours) to leach the salt and create pore structures. The scaffolds were soaked in 75% (vol/vol) ethyl alcohol overnight, washed three times with PBS, and coated with fibronectin (10 ng/ml) for 3 hours. Chondrogenically-committed cells were seeded onto porous scaffold and cultured for 3 weeks in chondrogenic differentiation medium supplemented with TGF-β1 (10 ng/ml).
(4.10 MB TIF)

## References

[1] Furman BD, Olson SA, Guilak F (2006). The development of posttraumatic arthritis after articular fracture.. J Orthop Trauma.

[2] LaPrade RF, Swiontkowski MF (1999). New horizons in the treatment of osteoarthritis of the knee.. Jama.

[3] Gillogly SD, Myers TH, Reinold MM (2006). Treatment of full-thickness chondral defects in the knee with autologous chondrocyte implantation.. J Orthop Sports Phys Ther.

[4] Lee J, Lee E, Kim HY, Son Y (2007). Comparison of articular cartilage with costal cartilage in initial cell yields, degree of dedifferentiation during expansion, and their redifferentiation capacity.. Biotechnol Appl Biochem.

[5] Prockop DJ (1997). Marrow stromal cells as stem cells for nonhematopoietic tissues.. Science.

[6] Sharma B, Williams CG, Khan M, Manson P, Elisseeff JH (2007). In vivo chondrogenesis of mesenchymal stem cells in a photopolymerized hydrogel.. Plast Reconstr Surg.

[7] Williams CG, Kim TK, Taboas A, Malik A, Manson P (2003). In vitro chondrogenesis of bone marrow-derived mesenchymal stem cells in a photopolymerizing hydrogel.. Tissue Eng.

[8] Song L, Baksh D, Tuan RS (2004). Mesenchymal stem cell-based cartilage tissue engineering: cells, scaffold and biology.. Cytotherapy.

[9] Kolf CM, Cho E, Tuan RS (2007). Mesenchymal stromal cells. Biology of adult mesenchymal stem cells: regulation of niche, self-renewal and differentiation.. Arthritis Res Ther.

[10] Caplan AI (2007). Adult mesenchymal stem cells for tissue engineering versus regenerative medicine.. J Cell Physiol.

[11] Vacanti V, Kong E, Suzuki G, Sato K, Canty JM (2005). Phenotypic changes of adult porcine mesenchymal stem cells induced by prolonged passaging in culture.. J Cell Physiol.

[12] Pelttari K, Winter A, Steck E, Goetzke K, Hennig T (2006). Premature induction of hypertrophy during in vitro chondrogenesis of human mesenchymal stem cells correlates with calcification and vascular invasion after ectopic transplantation in SCID mice.. Arthritis Rheum.

[13] De Bari C, Dell'Accio F, Luyten FP (2004). Failure of in vitro-differentiated mesenchymal stem cells from the synovial membrane to form ectopic stable cartilage in vivo.. Arthritis Rheum.

[14] Nagai A, Kim WK, Lee HJ, Jeong HS, Kim KS (2007). Multilineage potential of stable human mesenchymal stem cell line derived from fetal marrow.. PLoS ONE.

[15] Thomson JA, Itskovitz-Eldor J, Shapiro SS, Waknitz MA, Swiergiel JJ (1998). Embryonic stem cell lines derived from human blastocysts.. Science.

[16] Amit M, Itskovitz-Eldor J (2002). Derivation and spontaneous differentiation of human embryonic stem cells.. J Anat.

[17] Koay EJ, Hoben GM, Athanasiou KA (2007). Tissue Engineering with Chondrogenically-differentiated Human Embryonic Stem Cells.. Stem Cells.

[18] Hwang NS, Varghese S, Elisseeff J (2008). Controlled differentiation of stem cells.. Adv Drug Deliv Rev.

[19] Hwang NS, Kim MS, Sampattavanich S, Baek JH, Zhang Z (2006). Effects of three-dimensional culture and growth factors on the chondrogenic differentiation of murine embryonic stem cells.. Stem Cells.

[20] Kramer J, Hegert C, Guan K, Wobus AM, Muller PK (2000). Embryonic stem cell-derived chondrogenic differentiation in vitro: activation by BMP-2 and BMP-4.. Mech Dev.

[21] Nakayama N, Duryea D, Manoukian R, Chow G, Han CY (2003). Macroscopic cartilage formation with embryonic stem-cell-derived mesodermal progenitor cells.. J Cell Sci.

[22] Hwang NS, Varghese S, Puleo C, Zhang Z, Elisseeff J (2007). Morphogenetic signals from chondrocytes promote chondrogenic and osteogenic differentiation of mesenchymal stem cells.. J Cell Physiol.

[23] Xu C, Inokuma MS, Denham J, Golds K, Kundu P (2001). Feeder-free growth of undifferentiated human embryonic stem cells.. Nat Biotechnol.

[24] Xu C, Jiang J, Sottile V, McWhir J, Lebkowski J (2004). Immortalized fibroblast-like cells derived from human embryonic stem cells support undifferentiated cell growth.. Stem Cells.

[25] Kim TK, Sharma B, Williams CG, Ruffner MA, Malik A (2003). Experimental model for cartilage tissue engineering to regenerate the zonal organization of articular cartilage.. Osteoarthritis Cartilage.

[26] Hwang NS, Varghese S, Zhang Z, Elisseeff J (2006). Chondrogenic differentiation of human embryonic stem cell-derived cells in arginine-glycine-aspartate-modified hydrogels.. Tissue Eng.

[27] Livak KJ, Schmittgen TD (2001). Analysis of relative gene expression data using real-time quantitative PCR and the 2(-Delta Delta C(T)) Method.. Methods.

[28] Farndale RW, Buttle DJ, Barrett AJ (1986). Improved quantitation and discrimination of sulphated glycosaminoglycans by use of dimethylmethylene blue.. Biochim Biophys Acta.

[29] Woessner JF (1961). The determination of hydroxyproline in tissue and protein samples containing small proportions of this imino acid.. Arch Biochem Biophys.

[30] Vats A, Bielby RC, Tolley N, Dickinson SC, Boccaccini AR (2006). Chondrogenic differentiation of human embryonic stem cells: the effect of the micro-environment.. Tissue Eng.

[31] Kim MS, Hwang NS, Lee J, Kim TK, Leong K (2005). Musculoskeletal differentiation of cells derived from human embryonic germ cells.. Stem Cells.

[32] Elisseeff J, Ferran A, Hwang S, Varghese S, Zhang Z (2006). The role of biomaterials in stem cell differentiation: applications in the musculoskeletal system.. Stem Cells Dev.

[33] Elisseeff J, Puleo C, Yang F, Sharma B (2005). Advances in skeletal tissue engineering with hydrogels.. Orthod Craniofac Res.

[34] Burdick JA, Anseth KS (2002). Photoencapsulation of osteoblasts in injectable RGD-modified PEG hydrogels for bone tissue engineering.. Biomaterials.

[35] Hern DL, Hubbell JA (1998). Incorporation of adhesion peptides into nonadhesive hydrogels useful for tissue resurfacing.. J Biomed Mater Res.

[36] Zandstra PW, Nagy A (2001). Stem cell bioengineering.. Annu Rev Biomed Eng.

[37] Kim JS, Ryoo ZY, Chun JS (2007). Cytokine-like 1 (Cytl1) regulates the chondrogenesis of mesenchymal cells.. J Biol Chem.

[38] D'Angelo M, Pacifici M (1997). Articular chondrocytes produce factors that inhibit maturation of sternal chondrocytes in serum-free agarose cultures: a TGF-beta independent process.. J Bone Miner Res.

[39] Solursh M, Reiter RS (1975). The enhancement of in vitro survival and chondrogenesis of limb bud cells by cartilage conditioned medium.. Dev Biol.

[40] Toh WS, Yang Z, Liu H, Heng BC, Lee EH (2007). Effects of culture conditions and bone morphogenetic protein 2 on extent of chondrogenesis from human embryonic stem cells.. Stem Cells.

[41] Hegert C, Kramer J, Hargus G, Muller J, Guan K (2002). Differentiation plasticity of chondrocytes derived from mouse embryonic stem cells.. J Cell Sci.

[42] Lutolf MP, Hubbell JA (2005). Synthetic biomaterials as instructive extracellular microenvironments for morphogenesis in tissue engineering.. Nat Biotechnol.

[43] Yang F, Williams CG, Wang DA, Lee H, Manson PN (2005). The effect of incorporating RGD adhesive peptide in polyethylene glycol diacrylate hydrogel on osteogenesis of bone marrow stromal cells.. Biomaterials.

[44] Varghese S, Hwang NS, Canver AC, Theprungsirikul P, Lin DW (2007). Chondroitin sulfate based niches for chondrogenic differentiation of mesenchymal stem cells.. Matrix Biol.

[45] Takahashi I, Onodera K, Sasano Y, Mizoguchi I, Bae JW (2003). Effect of stretching on gene expression of beta1 integrin and focal adhesion kinase and on chondrogenesis through cell-extracellular matrix interactions.. Eur J Cell Biol.

