# Supplementary figures and images for: Derivation of Chondrogenically-Committed Cells from Human Embryonic Cells for Cartilage Tissue Regeneration
Source: PLoS One. 2008 Jun 25;3(6):e2498. doi: 10.1371/journal.pone.0002498 (PMC2423617; doi:10.1371/journal.pone.0002498)

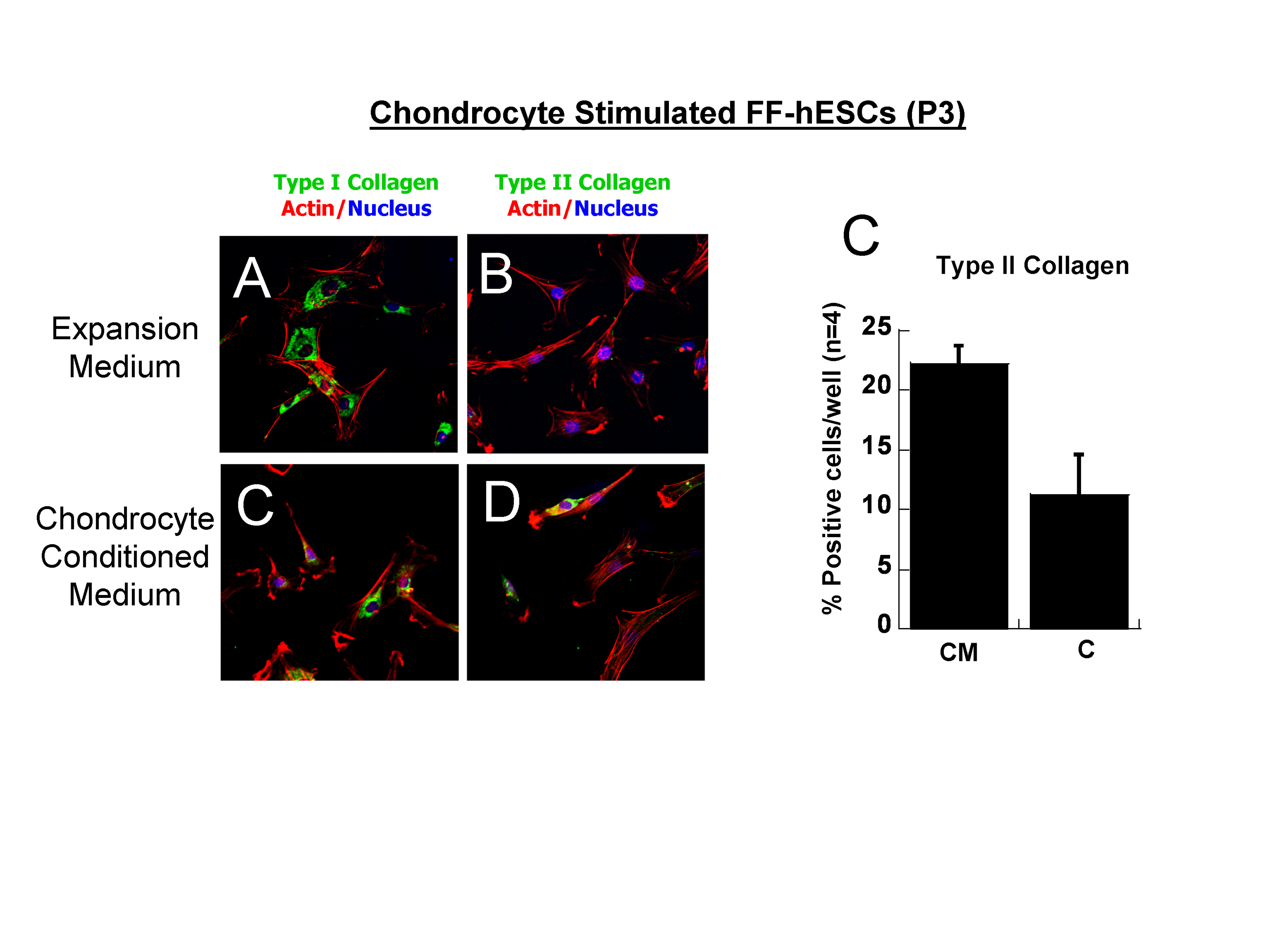

Supplement: Figure S1 — Chondrogenically-committed cells dedifferentiated upon initial expansion. At P3, chondrogenically-committed cells mainly expressed type I collagen (A) and type II collagen (B) was minimally detected when cultured with expansion medium (DMEM/FBS 10%). However, expansion of the cells in chondrocyte conditioned medium resulted in greater number of type II collagen positive cells (C, D). (E) Quantification of type II collagen positive cells that were expanded with either chondrocyte-conditioned medium (CM) or control expansion medium (C). (2.30 MB TIF) [file pone.0002498.s001.tif]

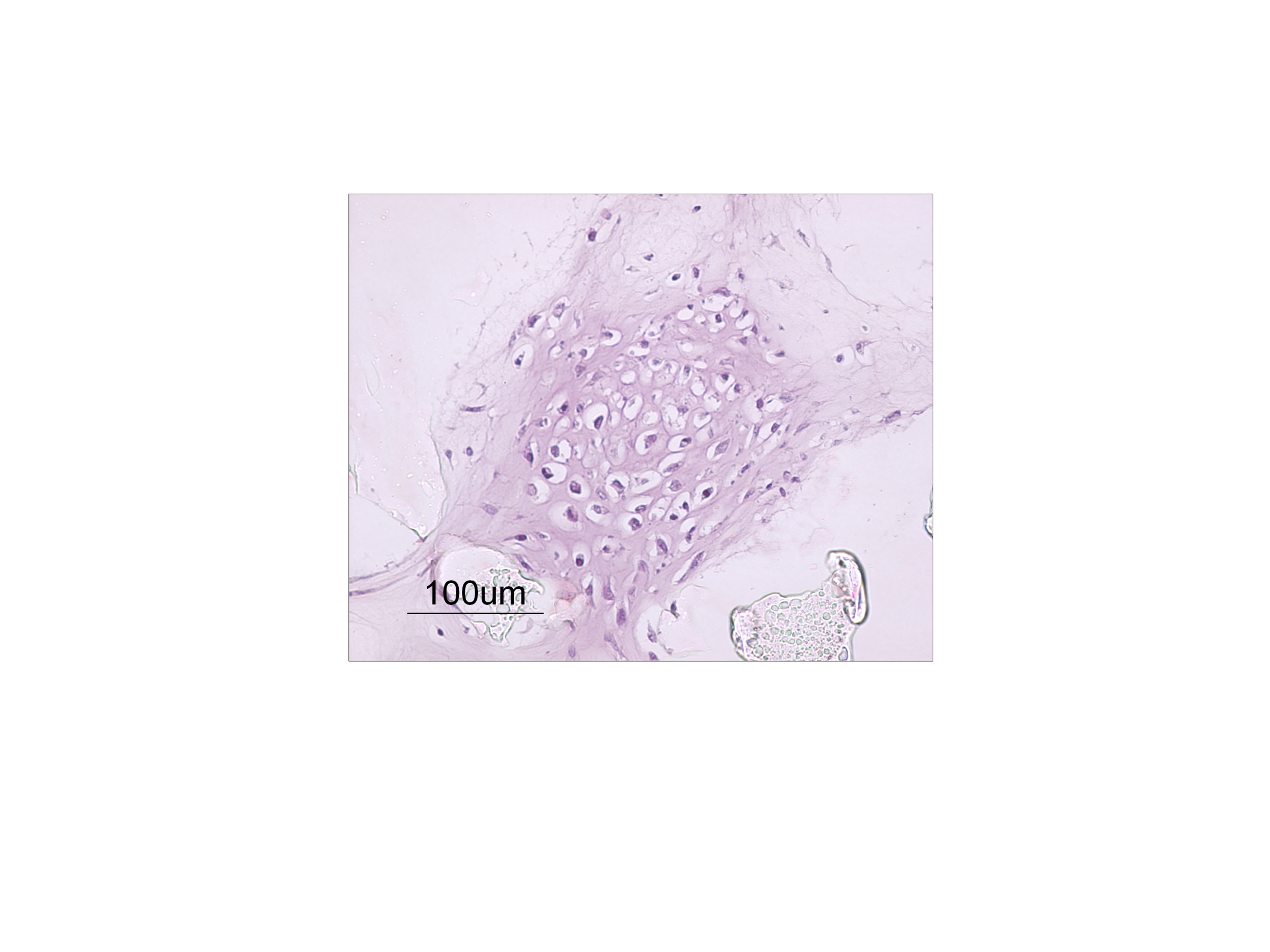

Supplement: Figure S2 — Safranin-O staining with eosin counter staining of in vitro cartilage tissue formation with chondrogenically-committed cells seeded on 3-dimensional porous scaffolds. Three dimensional porous scaffold composed of poly-(L-lactic acid) (PLLA) and poly-(lactic-glycolic acid) (PLGA) were fabricated by initially dissolving PLLA/PLGA 1:1 in chloroform to yield a solution of 5% (wt/vol) polymer. 0.25 ml of polymer solution was loaded into molds packed with 0.4 g of sodium chloride particles. The solvent was allowed to evaporate overnight and the sponges were subsequently immersed for 12 h in distilled water (changed every 2 hours) to leach the salt and create pore structures. The scaffolds were soaked in 75% (vol/vol) ethyl alcohol overnight, washed three times with PBS, and coated with fibronectin (10 ng/ml) for 3 hours. Chondrogenically-committed cells were seeded onto porous scaffold and cultured for 3 weeks in chondrogenic differentiation medium supplemented with TGF-β1 (10 ng/ml). (4.10 MB TIF) [file pone.0002498.s002.tif]
